# No red cell alloimmunization or change of clinical outcome after using fresh frozen cancellous allograft bone for acetabular reconstruction in revision hip arthroplasty: a follow up study

**DOI:** 10.1186/1471-2474-13-187

**Published:** 2012-09-25

**Authors:** Falk Mittag, Matthias Straub, Richard Schäfer, Torsten Kluba, Ingmar Ipach

**Affiliations:** 1 Department of Orthopaedic Surgery, University Hospital Tuebingen, Hoppe-Seyler-Str 3, Tuebingen, 72076, Germany; 2 Institute of Clinical and Experimental Transfusion Medicine, University Hospital Tuebingen, Otfried-Mueller-Str 4/1, Tuebingen, 72076, Germany

**Keywords:** Acetabular revision, Allograft bone, Remodeling, Alloimmunization, AB0, Rhesus

## Abstract

**Background:**

Possible immunization to blood group or other antigens and subsequent inhibition of remodeling or incorporation after use of untreated human bone allograft was described previously. This study presents the immunological, clinical and radiological results of 30 patients with acetabular revisions using fresh frozen non-irradiated bone allograft.

**Methods:**

AB0-incompatible (donor-recipient) bone transplantation was performed in 22 cases, Rh(D) incompatible transplantation in 6 cases. The mean follow up of 23 months included measuring Harris hip score and radiological examination with evaluation of remodeling of the bone graft, implant migration and heterotopic ossification. In addition, all patients were screened for alloimmunization to Rh blood group antigens.

**Results:**

Compared to the whole study group, there were no differences in clinical or radiological measurements for the groups with AB0- or Rh(D)-incompatible bone transplantation. The mean Harris Hip Score was 80.6. X-rays confirmed total remodeling of all allografts with no acetabular loosening. At follow up, blood tests revealed no alloimmunization to Rh blood group donor antigens.

**Conclusions:**

The use of fresh frozen non-irradiated bone allograft in acetabular revision is a reliable supplement to reconstruction. The risk of alloimmunization to donor-blood group antigens after AB0- or Rh-incompatible allograft transplantation with a negative long-term influence on bone-remodeling or the clinical outcome is negligible.

## Background

Aseptic loosening is the most common long-term complication in total hip arthroplasty. Revision of the failed acetabular component remains challenging due to migration of the implant during loosening and procedures to remove the primary implant often result in an extensive loss of pelvic bone. Bone grafting combined with insertion of a revision acetabular component is an established method to restore pelvic bone stock [1-4]. Because of its limited availability and poor quality in elderly patients the use of an autogenous graft is often not feasible. Therefore, allografts are utilized in most acetabular revisions. Regardless of whether treated (chemical, freeze dried, irradiated) or fresh-frozen non-irradiated allografts are used, the clinical outcome is usually good [5-7]. We have been using fresh frozen untreated allografts from our own bone bank in revision acetabular hip arthroplasty for decades with good results. Nevertheless, immunization to blood group antigens or other antigens and subsequent possible inhibition of long-term remodeling or incorporation of the transplanted bone is mentioned as an argument against the use of fresh frozen non-irradiated allografts [8,9].

The purpose of this study was to evaluate whether allografting of AB0- and Rh-incompatible patients (donor-recipient) leads to recipient-alloimmunization with proof of irregular erythrocyte antibodies (Rh system). In addition, clinical and radiological findings should be observed in the postoperative course.

## Methods

### Graft extraction

Femoral head bone grafts were obtained from donors through total hip arthroplasty. The grafts were not treated, immediately double packed and stored at – 80°C at our local bone bank. Besides blood group determination (AB0 and Rhesus) donors were screened for infectious diseases (HIV, Hepatitis B and -C, Syphilis) before and at least six weeks after surgery according to the local guidelines for operating a bone bank.

### Patients

We retrospectively reviewed 30 patients (13 males, 17 females). The study was performed in compliance with the Helsinki Declaration and approved by the local Ethics Committee (Nr. 254/2010BO2, University Tuebingen, Germany). Between 2006 and 2010 all included patients received fresh frozen cancellous allograft bone from our bone bank during acetabular revision at our institution by the corresponding author (T.K.). Acetabular defects were determined from preoperative radiographs and the intraoperative assessment using the classification introduced by Paprosky et al. [10]. Type I defects were present in 8 hips (26.7%), type II A in 9 (30%), type II B in 3 (10%), type II C in 3 (10%), type III A in 2 (6.7%), type III B in 3 (10%) and type IV with complete pelvic discontinuity in 2 (6.7%). The amount of impacted bone material was determined by the size of the defect. AB0 incompatible (donor-recipient) bone transplantation was performed in 22 cases. 6 Rh(D) negative patients received bone from Rh(D) positive patients. In most cases, revision components were implanted (Burch-Schneider reinforcement ring or Mueller ring, Zimmer GmbH, Switzerland) for acetabular reconstruction. The average age at the time of surgery was 71 years (range 48 to 90).

### Follow up

All patients were screened for alloimmunization to Rh blood group antigens (D, C, c, E, e) with a minimum clinical and radiographic follow-up of 6 months (mean 23 months). We did not screen for further blood group antigens.

Clinical assessments were evaluated according to the criteria of the Harris Hip Score including scoring of pain, walking and mobility of the revised hip [11].

Radiological evaluation was performed after 7 days, 6 weeks and at the time of study-related follow up at least 6 months after surgery. The acetabular index and horizontal and vertical migration of the acetabular component were measured [12]. Acetabular component loosening was defined if the sum of horizontal and vertical migration was ≥ 5 mm, if the change in the acetabular index was ≥ 5° or if there was a progressive radiolucent line ≥ 1 mm around the whole acetabular component [13]. The Brooker-classification was used for determination of heterotopic ossification [14]. Remodeling of the allograft was measured on the basis of appearance of trabecular remodeling within the graft.

### Statistical analysis

The paired *t*-test and Pearson´s chi square test were used for intra-group analysis. The Pearson correlation coefficient was calculated to measure the dependence between the different variables. A p-value ≤ 0.05 was considered significant.

## Results

### Alloimmunization

AB0 incompatible (donor-recipient) allograft transplantation was performed in 22 cases, Rhesus(D)-incompatible transplantation in 6 of 30 cases. No antibodies to donor blood-antigens were found in any patient in the Rhesus-system (Dd, Cc, Ee) during follow up. Especially Rh(D) incompatible transplantation did not lead to a detectable alloimmunization. We also found no differences in clinical or radiological measurements for these groups (Table 1).

**Table 1:** Study groups

	**AB0-incompatible transplantation**	Rh(D)-incompatible transplantation*	**All patients**
Number of patients (hips)	22	6	30
Gender (male:female)	8:14	1:5	13:17
Mean age in years (range)	71 (48 to 90)	66 (52 to 78)	71 (48 to 90)
*Acetabular revision component (number of hips)*			
Burch-Schneider ring	7	1	11
Müller ring	13	4	15
Standard cup	2	1	4
Mean follow up in month (range)	21 (6 to 55)	28 (14 to 38)	23 (6 to 63)
*Follow up*			
Alloantibodies to donor (Rh system**)	none	none	none
Mean Harris Hip Score (range)	79.5 (53 to 100)	86.3 (72 to 100)	80.6 (43 to 100)
Acetabular component tilting in ° (range)	1.37 (0.1 to 3.3)	1.00 (0.5 to 2.1)	1.29 (0 to 4,4)
Horizontal migration in mm (range)	2.17 (0.1 to 6.0)	2,27 (1.5 to 3.4)	2,01 (0.1 to 4.5)
Vertical migration in mm (range)	1.27 (0 to 4.2)	1.13 (0 to 2.1)	1,44 (0 to 4.2)
Graft remodeling rate (%)	100	100	100

* Rh(D)-negative patients received bone from Rh(D)-positive donors.

** Screening for antibodies against 5 Rhesus antigens (D, C, c, E, e).

### Clinical and radiological findings

**Figure 1:**
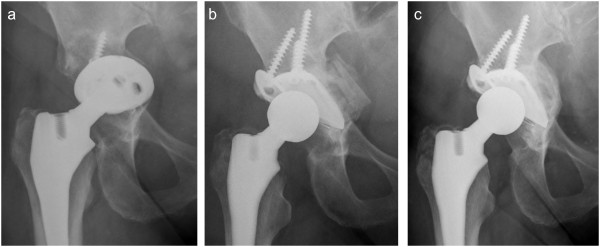
**a. Preoperative anteroposterior radiograph of a 72 year old woman, 13 years after total hip replacement, showing aseptic loosening of the acetabular component with migration into the small pelvis (type III B). b.** 7 days after acetabular revision using a Mueller ring and fresh frozen non-irradiated allograft bone. Note the distinguishable allograft bone chips medial of the ring. A polyethylene cup was cemented into the ring. **c.** 28-month follow up radiograph showing the implants to be unchanged with complete remodeling of the allograft bone and homogeneous trabeculation.

The revision rate of the entire study group of 30 patients was 3.3% due to a superficial septic complication in one patient after an AB0- and Rh-compatible allograft transplantation. All 30 acetabular components were still in place at time of follow up. The mean Harris-Hip-Score at the latest follow up was 80.6 points (range 43 to 100). Significant acetabular component tilting > 5° (range 0° to 4.4°), horizontal migration ≥ 5 mm (0.1 to 6.0 mm) or vertical migration ≥ 5 mm (0 to 4.2 mm) was found in one case. All allografts remodeled with homogeneous trabeculation and no radiolucent lines at the host-allograft interface (Figures 1). Periacetabular heterotopic ossification was found in 6 cases (20%): 5 patients with grade II and 1 patient with grade III. However, in 5 of 6 cases, preoperative radiographs revealed heterotopic ossification of a similar grade. There was no correlation between increasing preoperative acetabular defects and Harris Hip Score at follow up (p = 0.46). Advanced age was negatively correlated with the Harris Hip Score, but did not reach statistical significance (R = −0.51, p = 0.21).

## Discussion

The best method of preparation and processing of bone allografts is still under discussion. We use fresh frozen allografts in our department with no further chemical or physical processing. The use of fresh frozen allografts became subject to the restrictive European Union Directive due to the concern of a possible transmission of an infectious disease or other illness [15]. A low risk of disease transmission remains in some cases which are described in the literature [16,17]. Sufficient donor-screening is therefore essential. Except for Rh(D)-negative females of childbearing age, fresh frozen allografts are commonly transplanted AB0- and Rh-incompatible.

In the present study we found no alloimmunization in Rh(D)-incompatible transplanted recipients. With respect to other clinically relevant antigens in the Rh system (C, c, E, e) no irregular antibodies could be detected in the recipients after transplantation. However, reviewing the literature, there are some rare cases of Rh(D)-alloimmunisation after bone grafting [18,19].

Concerning the AB0 system, the human body always naturally contains antibodies against the other blood group antigens (except genotype AB). Therefore, the detection of anti-A or anti-B alloantibodies cannot be regarded as proof of a possible alloimmunization after AB0 incompatible transplantation. However, AB0 incompatible transplantation might cause an increase of the anti-A or Anti-B titer (boostering) [20]. Due to the retrospective design of our study, we could not investigate a possible boostering of anti-A or Anti-B alloantibodies after AB0-incompatible transplantation of bones. Stassen et al. found no irregular antibodies in patients before and after transplantation of frozen allogeneic bone in orthopaedic or maxillo-facial surgery [21].

Despite the fact that we found no antibodies and according to the current standards, we still recommend transplanting only Rh(D)-negative bones to Rh(D)-negative women of childbearing age. For all other patients, our study confirms that blood-group compatible transplantation of fresh frozen allografts is not necessary in revision hip arthroplasty.

To minimize the risks of infection, allografts could be sterilized by irradiation and chemical or physical treatment. This treatment could result in a destruction of the bone matrix and subsequent reduction in strength affecting long-term graft incorporation [22]. Despite this consideration, studies show good mid- and long-term results of treated bone grafts in revision hip arthroplasty [1-7,23-26].

Our study revealed 100% graft remodeling and only one case of significant acetabular component migration (6 mm migration with no clinical symptoms, no surgical revision necessary) at a mean follow up of 23 months. We found radiological evidence of good allograft trabeculation as a sign of complete remodeling and integration into the recipient bone structure, even after 6 months. This rapid remodeling rate has also been demonstrated in several other studies [23,24]. We have to consider, that complete remodeling does not mean complete incorporation of the graft. This could only be confirmed by biopsy and histological examination.

Acetabular reconstruction using a revision implant and allograft bone for reconstructing pelvic bone stock is a reliable method of managing acetabular defects. The question remains whether to use treated or untreated allografts in hip revision surgery as both show good clinical results. Advantages of untreated fresh frozen non-irradiated allografts are their cost effectiveness, supposed better biological quality and availability in a local bone bank. A disadvantage is the slightly increased risk of disease transmission which can be minimized by sufficient donor-screening and sterile handling.

## Conclusions

We conclude that the risk of alloimmunization against blood group antigens after AB0- or Rh-incompatible transplantation of bone with an influence on bone-remodeling or the clinical outcome is very low.

## Competing interests

The authors declare that they have no competing interests.

## Authors` contributions

Each author has made substantive intellectual contributions to this study: FM: participated in collecting data and study design, drafted the manuscript. MS: participated in collecting data. RS: participated in study design (immunologic research), manuscript revision. TK: participated in study design, performed the acetabular revisions, manuscript revision. IP: participated in study design, manuscript revision. All authors read and approved the final manuscript.

## Pre-publication history

The pre-publication history for this paper can be accessed here:

http://www.biomedcentral.com/1471-2474/13/187/prepub
